# The influence of prediction on bilingual language production: evidence from semantic classifier congruency

**DOI:** 10.1007/s00426-025-02204-2

**Published:** 2025-11-11

**Authors:** Jing Tong, Iring Koch, Andrea M. Philipp

**Affiliations:** Institute of Psychology, RWTH Aachen University, Jaegerstr. 17-19, Aachen, 52066 Germany

## Abstract

This study aims to investigate if predictions can influence subsequent language production in monolingual and bilingual situations. The possibility to correctly predict an upcoming response was operationalized by means of semantic classifier congruency. Participants were instructed to name a picture (e.g., firemen) after seeing or hearing a semantically congruent vs. incongruent classifier (e.g., a crew of). In Experiment 1 with English monolinguals, better performance was observed in semantically congruent trials (a crew of firemen) than in semantically incongruent trials (a packet of ants). In Experiments 2 and 3 with Chinese-English bilinguals, this semantic classifier congruency effect was replicated, but the effect differed in size as a function of the language (larger for L1 Chinese than for L2 English). Additionally, bilingual language control was influenced as the language-switch cost was smaller in congruent than in incongruent trials. Together, these findings suggest that prediction influences both L1 and L2 language production and that this facilitation of language production through prediction had a further impact on language control during language switching.

**Supplementary Information:**

The online version contains supplementary material available at 10.1007/s00426-025-02204-2.

## Introduction

Since the cognitive revolution in psychology kicked off in the 1950 s, it has become widely accepted that the mind functions as an information-processing system, a mechanism that enables humans to perceive and transform information from the surrounding world (Greenwood, 2015; Wickens, 2021). By learning the statistical regularities of incoming information, the brain gradually develops predictive mechanisms that help individuals to navigate more effectively in the real world (Rao & Ballard, 1999). Such prediction is also important in the domain of language processing (Ryskin & Nieuwland, 2023). Indeed, during daily conversation, prediction could be activated to keep up with successful communication. While previous studies have focused on how the predictability of upcoming input influences language processing in monolinguals and bilinguals (e.g., Grisoni et al., 2021; Pulvermüller & Grisoni, 2020), less attention has been given to how prediction itself affects language control in bilingual context. In the current study, we aim to investigate the influence of prediction triggered by a highly constrained context on bilingual language control.

### Language switching and Language control in bilinguals

The language-switching paradigm is commonly used to explore language control in bilingual language production (cf. Meuter & Allport, 1999; for a review, see Declerck & Philipp, 2015). In this paradigm, participants typically name pictures or digits in one language or another as a function of a cue (e.g., a color or a flag). Results showed that participants need more time to switch from one language to another as compared to repeating the same language in two successive trials, demonstrating a switch cost in language production (e.g., Christoffels et al., 2007; Heikoop et al., 2016; Kroll et al., 2008; Meuter & Allport, 1999; Philipp et al., 2007; Peter et al., 2014).

According to the inhibitory control model (Green, 1998), a language-switch cost arises as a function of an inhibitory language-control mechanism. That is, in order to produce a word in the intended language, words (i.e., lemmas) of the competing language have to be inhibited. A language-switch trial, thus, suffers both from persisting inhibition of the previously irrelevant language that now becomes relevant and from persisting activation of the previously relevant language that now becomes irrelevant. Some studies found asymmetrical switch cost, showing smaller switch cost in the second language (L2) than in the first language (L1; Costa & Santesteban, 2004; Liu et al., 2016; Meuter & Allport, 1999; Philipp et al., 2007). To explain this asymmetry, it is often assumed that language inhibition is proportional to the degree of language activation (cf. Green, 1998; for a review, see Declerck & Koch, 2023). For unbalanced bilinguals, L1 is more dominant than L2 and thus becomes activated to a higher degree – so that more inhibitory language control would be needed. However, a recent meta-analysis demonstrated that this pattern is not consistently observed across studies (see Gade et al., 2021), highlighting the need for further empirical evidence in this area.

Most studies using a language-switching paradigm primarily focused on bilingual language production on the level of single-words (i.e., mainly digit naming or picture naming, for exceptions, see, e.g., Sánchez et al., 2022; Tarlowski et al., 2013). Moreover, in most studies, such word-production tasks were performed without a linguistic context. In contrast, language production in our daily lives often is part of a conversation and, thus, also depends on what has been produced by our interaction partner. In other words, language production might be triggered by an external input (i.e., linguistic input that we perceive and comprehend), highlighting the need to question how such external input influences language production during language switching. To understand this process, it is crucial to consider that this external input is not only perceived but also analyzed by the statistical regularities in our predictive mind, suggesting that prediction mechanisms may be activated even before language production begins.

Importantly, predictions that arise from processing an external input could influence subsequent language production on several levels: On the one hand, the language in which we perceive the external input can influence the language used for language production (for evidence see, e.g., Gambi & Hartsuiker, 2016; Liu et al., 2018). On the other hand, the external input also constrains syntactic and semantic options. That is, processing the beginning of a phrase or a sentence restricts how this sentence can be correctly continued or completed in both syntactic and semantic terms. In this study, we are mainly interested in the role of predictions on a semantic level (although control variables with respect to the syntactic level will be addressed in Experiment 1). More precisely, in the present study the external input will always be presented in the same language as the production task (although the language switches between trials in Experiments 2 and 3) so that predictability is only manipulated in terms of a high vs. low constraining semantic context (i.e., the language of the to-be-produced word can be fully predicted by the language of the external input but the predictability of the semantic concept is high vs. low).

Interestingly, a previous study Declerck et al. (2015a, b) already explored the influence of language and/or concept (i.e., semantic) predictability on language control in a language-switching paradigm. In their study, participants had to memorize a language and/or a concept sequence so that in different conditions either nothing, the language, the concept or both was fully predictable. The language-switch cost in this study was markedly reduced only when both the language and the concept (i.e., the specific response in this trial) were predictable. Thus, this study provides evidence that, when the language is predictable (as it will be the case in our study), knowing which word to produce has an impact on language control. However, it is also important to keep in mind that participants in this study were explicitly instructed to memorize a specific concept sequence so that the concept of the word was either fully predictable or not predictable at all - which clearly differs from predictions based on processing external input, creating a high vs. low constraining context.

### Prediction in Language comprehension and production

Word processing is affected by the preceding words that provide a certain level of predictive contextual information. In a monolingual setting, predictions based on a highly constraining context have been validated to affect language comprehension (Grisoni et al., 2021; Lelonkiewicz et al., 2021; Kim et al., 2023; Leon-Cabrera et al., 2019; Martin et al., 2018; Ness & Meltzer-Asscher, 2018). Further, predictions in a monolingual setting were also shown to affect language production. In a study by Grisoni et al. (2024), participants had to complete a sentence in a high or low constraint context (i.e., a sentence context that either allowed or did not allow a specific prediction of the final word). The authors observed an anticipatory brain activity (defined by the mean ERP amplitude during the last 200 ms before the speech onset) in a high constraint context. The observed facilitation of language production in this context, thus, showed evidence for an influence of prediction on subsequent language production in a monolingual situation.

As our study primarily focuses on bilinguals’ language processing, we are also interested in exploring research that investigates whether prediction occurs and influences language processing in a later-learned language (i.e., L2; for reviews, see Kaan, 2014; Schlenter, 2023). The research pattern is relatively mixed as some studies (Ito et al., 2017; Mitsugi & Macwhinney, 2016) did not find evidence of predictions during L2 processing, whereas other studies found an effect of predictions also in L2 (Chun & Kaan, 2019; Dijkgraaf et al., 2017; Foucart et al., 2014). Yet, Martin et al. (2013) suggested a weaker predictive ability in L2 processing than in L1 processing (see also Momenian et al., 2024). Similarly, Dijkgraaf et al. (2017) demonstrated predictive eye movements in Dutch-English bilinguals and English monolinguals when listening to a highly constrained English sentence. However, these predictive eye movements appeared later in the second language of bilinguals than in monolinguals.

So far, rich evidences have demonstrated that prediction influences language processing. This naturally raises the question of how this process looks like. Some researchers have proposed that prediction happens through the use of the production system (Martin et al., 2018; Dell & Chang, 2014; Federmeier, 2007; Kuperberg & Jaeger, 2016; Lelonkiewicz et al., 2021; for reviews, see Pickering & Garrod, 2007, 2013; Pickering & Gambi, 2018). Specifically, when listening to other’s utterance or when reading a text, we will predict the next words by involving our own language production system. Through prediction by the production system, the linguistic levels of words (i.e., meaning-, grammar- and sound-related representation) for the following input are activated either sequentially (Pickering & Gambi, 2018) or in parallel (Pickering & Strijkers, 2024). Although these studies did not specify the finer-grained aspects of prediction, such as the number of concepts/words being activated, previous research on language comprehension (DeLong et al., 2005; Wlotko & Federmeier, 2012) suggests that comprehenders generate predictions by evaluating the likelihood of various possible outcomes. These probabilities are dynamically adjusted as bottom-up input either confirms or contradicts the predictions during language processing.

Furthermore, with respect to an influence on language production, Staub et al. (2015) showed that predictive processes in language production can be explained using an activation-based race model. This model proposes that in highly constrained contexts, multiple potential responses are activated simultaneously. These responses then independently “race” to reach a response threshold, with the first to do so determining the final output (i.e., language production). These studies indicate that prediction can generate a range of possibilities. The conclusion that prediction activates such a range of possibilities is also supported by research showing that prediction generally activates animacy features, rather than specific words (Wang et al., 2020).

### Prediction via classifier processing: semantic classifier congruence vs. incongruence

In this study, we aim at exploring the influence of prediction on subsequent language production by using classifier-noun phrases. More specifically, participants will read (Experiments 1 and 2) or hear a classifier (e.g., a bowl of) and produce a noun based on a picture presented on a screen. That is, a classifier was employed to establish a highly constrained context.

In general, classifiers can be used to further add information to a subsequent noun as for example with respect to the form of existence (e.g., a bowl of noodles). Importantly, the combination of classifiers and nouns is not arbitrary. Some classifiers are conventionally associated with specific nouns, and certain nouns are typically paired with particular classifiers (Lehrer, 1986). As studies have shown that language learners are sensitive to the transitional probabilities between adjacent elements in language (Romberg & Saffran, 2010) and children begin to make predictions once they have acquired sufficient language experience (Rabagliati et al., 2016), this statistical regularity in the combination of classifier and nouns can be acquired during language learning. Thus, we suppose that classifiers establish a highly constrained context, and processing them activates a prediction process.

In the context of the present study, we refer to conditions in which a classifier has to be completed with a highly predictable noun as sematic classifier congruence whereas a condition in which a noun that is less predictable by a classifier is referred to as semantic classifier incongruence (e.g., a bowl of noodles is a semantically congruent classifier-noun phrase whereas a bowl of students is semantically incongruent).

Previous studies have shown better performance in a congruent than in an incongruent classifier condition when participants perform a phrase-production task combined with a picture-word interference paradigm (i.e., classifier + noun; Huang & Schiller, 2021; Wang et al., 2025a, b; Zhang & Liu, 2009). Additionally, some researchers further investigated if processing a classifier could also lead to the prediction and a corresponding activation of semantically congruent conceptual/lexical representations. For example, using a visual world paradigm, Huettig et al. (2010) presented Chinese classifier-noun phrases to Chinese participants. Results showed that when participants heard the classifier (but not heard the noun yet), they looked to a semantically congruent picture more frequently than to a distractor (i.e., semantically incongruent) picture. With a very similar design, Mitsugi (2020) generalized this finding to native Japanese speakers and L2 Japanese learners. These findings clearly suggest that hearing a classifier can already activate semantically congruent conceptual/lexical representations. This also implies that participants make predictions about the following words while processing a classifier, even in a language that is not their native language.

In the current study, we focus on Chinese classifiers and their corresponding English translation equivalents. Although English (in contrast to Chinese) is typically considered to be a non-classifier language, classifiers in Chinese also convey semantic features similar to those in English (e.g., measure classifier like “a box of”, arrangement classifier like “a row of”, and collective classifier like “a nest of”; Allan, 1977, 1978).

### Current study

The aim of the present study was to explore the effect of prediction that can be built during processing an external input on language production (i.e., picture naming). Prediction thereby was operationalized through semantic classifier congruence vs. incongruence, with classifiers representing a high vs. low constrained context. To achieve this, participants were presented with a classifier and then had to name a picture, so that a classifier-noun pair was used in each trial. We assumed that processing a classifier will lead participants to predict the subsequent naming response with respect to both the language (which was always the same as the classifier language) and the semantic meaning of the response (which should be semantically congruent to the classifier). Yet, the classifier and the to-be-produced noun could be semantically congruent or incongruent in the present study. In case of a semantically congruent classifier-noun combination, the to-be produced noun was more likely predicted during the processing of the classifier than in semantically incongruent combinations. Thus, participants should show a better performance in semantically congruent classifier-noun trials as compared to semantically incongruent classifier-noun trials (i.e., a semantic classifier congruency effect).

A novel aspect of the current study hereby is that we explored the effect of prediction by means of classifier (in)congruence for both English monolingual speakers (Experiment 1) and Chinese-English bilinguals (Experiments 2 and 3). By doing so, we intend to provide evidence that semantic classifier congruence as a marker of prediction also can be demonstrated in English (Experiment 1), which was never tested before. Once semantic classifier congruence was established in English, we would extend the approach to a bilingual (i.e., Chinese-English) context. In Experiments 2 and 3, we examined the influence of prediction on bilingual language control in a language-switching setting. In these experiments, we also expected a semantic classifier congruency effect, which however might be stronger in L1 (Chinese) than L2 (English), revealing a weaker influence of prediction in L2 than in L1 (cf. Martin et al., 2013; Momenian et al., 2024). Furthermore, when prediction influences language control, the language-switch cost (referring to a language switch vs. language repetition in the picture-naming task) should be affected by the higher predictability in semantically congruent classifier-noun phrases, as compared to incongruent ones (in which the to-be produced noun was less predictable). Based on the findings by Declerck et al. (2015a, b) we reasoned that language-switch costs should be reduced in trials with congruent classifier-noun phrases as only in these trials both the language (based on the classifier language) and the to-be-produced noun could be predicted based on classifier processing.

## Experiment 1

In Experiment 1, we specifically tested whether there is a semantic classifier congruency effect in English. This effect will be indicated by better performance when classifier and noun are semantically related (i.e., congruent, e.g., a crew of firemen) than when they are unrelated (i.e., incongruent, e.g., a packet of firemen).

For this experiment, it is important to point to a critical difference between syntactic aspects in Chinese and English. Specifically, in term of the counting systems, English distinguishes between count nouns and mass nouns (i.e., countable nouns like one fireman, two firemen vs. nouns without a specific plural form like snow), whereas Chinese lacks this syntactic distinction in nouns (Lyons, 1977). Since previous studies have demonstrated that syntactic and semantic processing are highly interactive during language processing (Humphries et al., 2006; Kim & Sikos, 2011), English-speaking participants in the present experiments may be further influenced by the syntactic match or mismatch between the classifier and the noun in the picture-naming task. For example, after processing the classifier “a crew of”, the to-be-produced noun should be firemen (i.e., plural term). If the picture indicating the to-be-produced word would show a single fireman, this could be perceived as a syntactic mismatch although semantically the fireman is congruent to the classifier. To additionally test the influence of a syntactic match vs. non-match between classifier and noun, we used two manipulations of the picture/word material in Experiment 1. On the one hand, half of the nouns used in Experiment 1 resembled mass nouns and the other half count nouns (variable: word type) and, on the other hand, the picture showed either one or two items of the same object (variable: picture type). We reasoned that if syntactic aspects play a role in our experimental set-up, the picture type should have a larger influence on count nouns (where “a crew of firemen” is syntactically more correct than “a crew of fireman”) than in mass nouns (in which no plural forms exist as in “a shovel of snow”). As these variables were included only to control for syntactic influences, the results will mainly serve as guidance for refining the picture stimulus in subsequent experiments.

### Method

#### Participants

Twenty-six native English speakers (11 females, 15 males; 23 right-handed; mean age: *M* = 24.50 years old, *SD* = 4.13) took part in this laboratory experiment. All had normal or corrected-to-normal vision and no history of neurological or psychological impairments or receiving treatment with any psychoactive medication. All procedures in our study were in line with the protocol of the ethics committee of the faculty of Arts and Humanity RWTH Aachen University. All participants signed written informed consent and data protection, and received money (i.e., 10 €/hour) as compensation.

#### Materials and apparatus

To choose the appropriate materials, a different group of 40 native English speakers rated English classifier-noun phrases on a five-point scale (1 = very uncommon, 5 = very common) in a first pre-test. Additionally, they suggested typical nouns for classifiers (i.e., sentence completion tasks “a spoon of….”). Based on the results of this pre-test, we selected 24 nouns and their most appropriate congruent and incongruent classifier as stimulus material for Experiment 1 (for the full set of classifier-noun pairs see Table 3). The nouns were selected in a way that 12 nouns could be used in a singular or plural version (i.e., count nouns) and 12 would be used in a neutral version only (i.e., mass nouns). As we slightly adapted the classifier-noun combinations based on the results of first pre-test, another novel group of 26 native English speakers rated English classifier-noun phrases on a five-point scale (1 = very uncommon, 5 = very common) in a second pre-test. A paired-sample t-test showed that there was a significant difference between congruent and incongruent classifier-noun combinations (4.66 vs. 1.62), *t*(25) = 12.55, *p* <.001, thus indicating that the selection of semantically congruent and incongruent classifier-noun phrase worked well.

In a next step, we selected 24 color pictures (7.5 cm × 7.5 cm) corresponding to the 24 selected nouns. Nineteen of them were chosen from the Multilingual Picture (MultiPic) databank (Duñabeitia et al., 2018) and the rest was adapted from free online images. We also choose additional 7 pictures (Duñabeitia et al., 2018) for practice trials and warm-up trials of each block. For each participant, half of the pictures (50% of them referring to mass and 50% to count nouns) included one item, the other half included two items. The number of items per picture/concept was counterbalanced across participants. All pictures were framed in red or blue color (counterbalanced across participants) to be consistent with the bilingual experiments (in which the color frame represent different language cues, see below). All classifiers were presented as written words and appeared in Times New Roman, size 64 (English).

The experiment was programmed and run using PsychoPy2 (Peirce et al., 2019), version 2021.2.3. Speech onset of the vocal picture naming responses was recorded with a voice key using a microphone. Speech errors were coded offline by an experimenter during the experiment.

#### Procedure

**Fig. 1:**
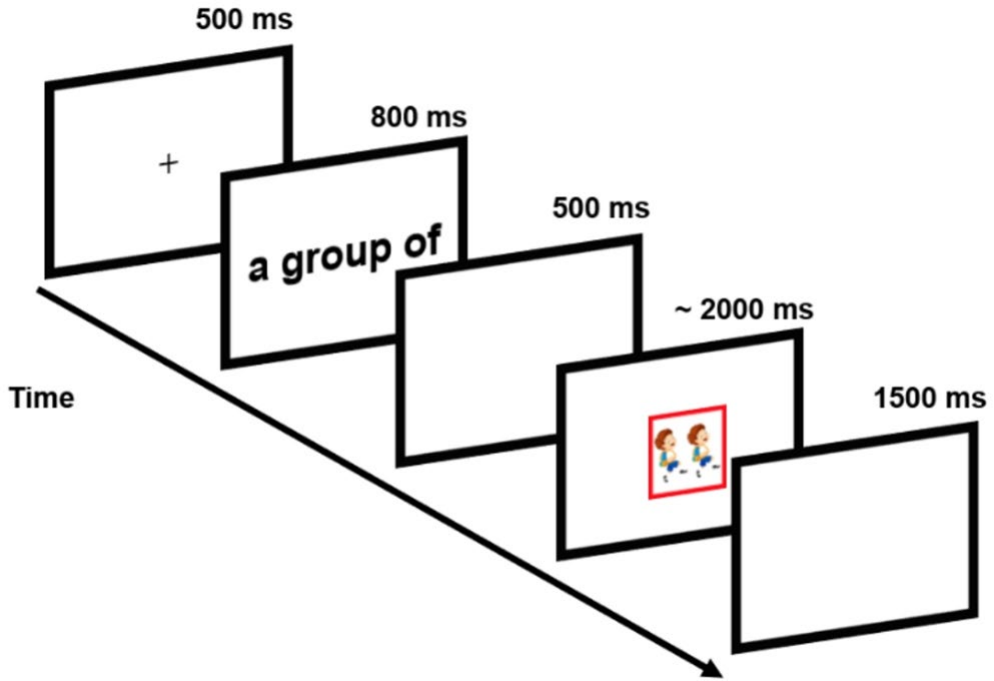
Experimental procedure of Experiments 1 and 2

In the picture-naming task, participants were asked to name the picture as fast and as accurately as possible (see Fig. 1). Each trial started with a fixation cross for 500 ms. After this time, the English classifier appeared visually for 800 ms followed by a 500 ms blank screen. Finally, a color-framed picture was presented. The picture would disappear once participants named the picture. If a participant did not respond within 2000 ms, the picture would disappear automatically. Then the screen turned blank for 1500 ms before the next trial started.

There were 10 blocks. Each block contained 48 trials and 2 warm-up trials. For 48 classifier picture name pairs, there were 24 pictures (see Table 3). Each picture was once paired with a semantically congruent and once with an incongruent classifier. Each classifier-picture pair appeared once in each block. In total, there were 8 conditions resulting from the combinations of three factors: semantic congruence (noun semantically congruent vs. incongruent to the classifier), word type (singular/plural vs. neutral), and picture type (picture presenting one vs. two items). The sequence of trials was pseudo-random to make the number of trials for each condition equal.

#### Design

We used a 2 × 2 × 2 within-subjects designs with the independent variables semantic congruence (noun semantically congruent vs. incongruent to the classifier), word type (count nouns vs. mass nouns), and picture type (one-item vs. two-items picture).

#### Data analysis

We used the function buildmer in the buildmer package (version 2.8; Voeten, 2019) within R (R Core Team, 2022) to analyze data and to construct a best fitting linear mixed-effect model. Model selection procedure started from the maximal model. If the maximal model did not converge, this function performed a backwards stepwise elimination until it did. After obtaining the best fitting model, we then using the lme4 package (version 1.1–32; Bates et al., 2014) to analyze the data.

The model with maximal structure included semantic congruence, word type and picture type as fixed effects, participants and items as random effects. Semantic congruence, word type, and picture type were included as by-subject slopes. Further, we considered semantic congruence as by-item random slope, which was justified by our design. However, in this experiment, word type was defined by an intrinsic property of the words, being either countable or mass nouns. Additionally, for each participant, we paired half of the countable nouns and half of the mass nouns with pictures presenting one item and the other half with pictures presenting two items (the combination of word and picture type was counterbalanced across participants). That is, word type and picture type varied between items within each participant. Since these two effects were between-items variables, we excluded them from by-item random slopes.

All fixed effects were two-level categorical predictors and coded as −0.5 and 0.5. For RT analysis, RTs were log-transformed and used as dependent variable (DV). We did not analyze accuracy further in Experiment 1, since it was close to ceiling (99.05%).

### Results

Error trials were coded by a research assistant in real time as follows: wrong word, wrong language or no response. The first two trials of each block (i.e., the two additional warm-up trials) and all trials with RT outside ± 3 z around the mean of all trials for each participant were excluded. For RT analysis, error trials, trials after an error response and “empty” trials caused by voice key errors were excluded. In the end, 4.26% of all trials were excluded from RT analysis.

#### Reaction time

The main effect of semantic congruence was significant, *β* = 0.03, *SE* = 0.01, *t* = 3.81, *p* =.003, with 22 ms faster responses in the trial in which the noun that was semantically congruent to the classifier than in those in which the noun was incongruent (727 ms vs. 749 ms).^1^ The interaction of word type and picture type was significant, *β* = 0.02, *SE* = 0.01, *t* = 2.88, *p* =.004. The data pattern shows that participants responded 10 ms faster for count nouns when the picture presented two items rather than one item (733 ms vs. 743 ms), whereas for mass nouns participants responded 5 ms faster when the picture presented one item rather than two items (735 ms vs. 740 ms). Yet, follow-up analyses on each word type in isolation showed neither a significant difference between the two picture types when the responding word was a count noun *(β* = 0.01, *SE* = 0.01, *z* = 1.34, *p* =.18), nor when it was a mass noun (*β* = − 0.01, *SE* = 0.01, *z* = −1.49, *p* =.14). There were no other significant main effects or interactions (all *ps* > 0.08; more details of the final model can be found in the supplementary materials Table S1).

#### Sensitivity analysis

We performed a post hoc sensitivity analysis for power analysis focusing on the main effect of interest. Power analysis was conducted based on simulating 100 random datasets using the *simr* package (Brysbaert & Stevens, 2018; Green & MacLeod, 2016; Green et al., 2016) in R (R Core Team, 2022). This package provides power estimates specifically for linear mixed-effects models. According to the simulation results, the power for the main effect of semantic congruence was between 96.38% and 100%, and the effect size for the main effect of semantic congruence was 0.036, indicating our design had enough power to detect the main effect of semantic congruence.

### Discussion

Experiment 1 revealed a semantic classifier congruency effect that replicates previous studies (Wang et al., 2006, 2025a, b; Huang & Schiller, 2021; Zhang & Liu, 2009). According to previous studies on Chinese speakers and Japanese speakers (Huettig et al., 2010; Mitsugi, 2020), we suppose that the classifier in English also led to an activation of semantically congruent concepts/words, facilitating corresponding naming responses.

Other than that, we observed that the semantic classifier congruency effect was not significantly modulated by word type or picture type. Thus, we are confident that the present results speak at least against a medium to large interaction effect of word or picture type and semantic congruence. Yet, given the present results (especially the interaction of word type and picture type), it is likely that syntactic features provided by the target picture (i.e., whether the corresponding noun had a singular/plural vs. a neutral form and how many items were presented) could play a small role in language production in classifier-noun phrases. It was obvious that the effect of semantic congruence was more prominent than any other effect. Nevertheless, in order to avoid effects based on numerus and syntactic features in the naming response, we always used two-item pictures for countable singular/plural nouns and one-item picture for non-countable mass nouns in our following experiments, in which we combined semantic classifier congruency with language switching in Chinese/English bilinguals.

## Experiment 2

In Experiment 2, we examined the semantic classifier congruency effect in a bilingual language-switching situation to observe if this effect differed between L1 and L2 and whether it interacted with language-switch cost. According to the result of Experiment 1 (cf. Huettig et al., 2010; Mitsugi, 2020), reading a classifier can co-activate the conceptual/lexical representations of semantically congruent nouns. Additionally, reading a classifier in one particular language may facilitate the following word production in this language. That is, we suppose that participants always will use the classifier to activate the language and semantically congruent concepts/words. In congruent trials, the naming response will then be facilitated by the pre-activation of both the language and the concept/word, whereas in incongruent trials, the language could be correctly pre-activated but the to-be-produced noun would be not as other concepts/nouns had been predicted based on the classifier.

As the language will be manipulated between trials, Experiment 2 can test for differences between the effect of prediction on language production in L1 and L2. Based on previous studies (Martin et al., 2013; Momenian et al., 2024) we expect a weaker influence of prediction in L2 than in L1. Further, we also suppose that prediction might have an effect language switching (i.e., switching the relevant language of the picture naming task between trials) and, thus, on bilingual language control.

### Method

#### Participants

Forty participants studying at RWTH Aachen University took part (19 females, 21 males; 39 right-handed; mean age: M = 24.18 years old, SD = 2.91). Chinese was their native language (L1) and English their second language (L2). Before the experiment, participants were asked to fill in a questionnaire about the age of L2 acquisition, the self-rated language skills in English (7-point scale: 1 = quite poor, 7 = highly proficient, see Table 1) and completed a proficiency online testing in a sentence completion task (http://www.itt-leipzig.de/static) for both Chinese and English. The results of a paired-sample t test showed a significant difference between the scores of L1 (90.58% accuracy) and L2 (54.23% accuracy), *t*(39) = 16.96, *p* <.001.

**Table 1:** Participants’ characteristics in experiment 2

Item	L2
AOA	7.27 (2.45)
Self-rating	
*Listening*	4.75 (0.83)
*Speaking*	4.55 (1.03)
*Reading*	5.25 (0.92)
*Writing*	4.33 (1.05)

*AOA* age of acquisition. The self-rated language skills were reported on a 7-point scale: 1 = “quite poor”, 7 = “highly proficient”. The value in the bracket indicates the standard deviation

#### Materials and apparatus

The materials and apparatus were identical to those used in Experiment 1 except that we changed some combinations of classifiers and pictures (see Table 4) based on the feedback and rating from Experiment 1.

#### Procedure

The procedure was similar to that used in Experiment 1. However, there were three changes in this experiment.

First, the picture type always syntactically matched with the classifier. That is, if the meaning of the classifier implied more than one item, the pictures would include two items (e.g., a group of students). If not, the pictures would present one item (e.g., a glass of honey).

Second, the participants were asked to use either Chinese or English for naming the picture according to a colored frame (red vs. blue, see Fig. 1). Each color represents one of the languages (i.e., L1 Chinese vs. L2 English). The color-language mapping was counterbalanced across participants. Please note that the classifier, as in Experiment 1, was always presented in the same language as the response had to be given in. Thus, the colored frame was an additional, explicit (but actually redundant) language cue that reminded participants which language to use.

Third, there were ten blocks with each block consisting of 2 warm-up trials and 48 formal trials. Each block for the formal trials contained the same 24 picture names in each language both in the congruent condition and the incongruent conditions. Six items appeared once in each condition (L1 repeat, L1 switch, L2 repeat, L2 switch) and each item could be assigned to a different condition across blocks. The sequence of trials was made pseudo-random to ensure an equal number of trials for each condition.

#### Design

We used a 2 × 2 × 2 within-subjects designs with the independent variables semantic congruence (noun semantically congruent vs. incongruent to the classifier), language (L1 vs. L2), and language sequence (repeat vs. switch).

#### Data analysis

Data analysis was the same as in Experiment 1. For the maximal model, semantic congruence, language and language sequence were fixed effects, participants and items were random effects. Semantic congruence, language and language sequence were included as a by-subject slopes and by-item slopes. All fixed effects were two-level categorical predictors and coded as −0.5 and 0.5. For RT analysis, RTs were log-transformed and used as dependent variable (DV). For accuracy analysis, response type (correct trials coded as 1; error trials coded as 0) was the dependent variable (DV).

### Results

For data exclusion, the same criteria as in Experiment 1 were applied in this experiment. In total, 10.72% of trials were excluded for RT analysis. For the accuracy analysis, we included correct trials and wrong response trials, but not trials with RT outside ± 3 z around the mean, trials after an error response and “empty” trials caused by voice key errors. In total, 7.69% of trials were excluded for accuracy analysis.

#### Reaction time

The main effect of semantic congruence was significant, *β* = 0.02, *SE* = 0.003, *t* = 5.94, *p* <.001, with 18 ms faster responses in trials in which the noun was semantically congruent to the classifier than in those in which the noun was incongruent (1046 ms vs. 1064 ms). The main effect of language was also significant, *β* = − 0.09, *SE* = 0.01, *t* = −8.88, *p* <.001, with faster responses in L2 than in L1 trials (1007 ms vs. 1103 ms). Finally, the main effect of language sequence was significant, *β* = 0.05, *SE* = 0.01, *t* = 5.14, *p* <.001, with faster responses in repeat trials than in switch trials (1032 ms vs. 1078 ms), showing a 46 ms language-switch cost.

The interaction of semantic congruence and language was significant, *β* = − 0.03, *SE* = 0.01, *t* = −3.92, *p* <.001. Follow-up analyses showed that participants were 31 ms faster in congruent than incongruent trials in L1 (1087 ms vs. 1118 ms), *β* = − 0.03, *SE* = 0.01, *z* = −6.92, *p* <.001, but only 5 ms faster in congruent than in incongruent trials in L2 (1005 ms vs. 1010 ms), *β* = − 0.01, *SE* = 0.01, *z* = −1.46, *p* =.14). This shows a clear semantic classifier congruency effect in L1 Chinese but no clear congruency effect in L2 English.

The interaction of language and language sequence was significant, *β* = 0.03, *SE* = 0.01, *t* = 3.99, *p* <.001. The data pattern indicates a smaller language-switch cost in L1 than in L2 (33 ms vs. 59 ms). Follow-up analyses indicated a significant switch cost in both L1 (1086 ms vs. 1119 ms), *β* = − 0.03, *SE* = 0.01, *z*= −3.45, *p* =.001, and L2 (978 ms vs. 1037 ms), *β* = − 0.06, *SE* = 0.01, *z* = −6.22, *p* <.001. There were no other interactions (*ps* < 0.09; more details of the final model could be found in supplementary materials Table S2).

#### Accuracy

The main effect of language was significant, *β* = 4.42, *SE* = 0.51, *z* = 8.69, *p* <.001, with higher accuracy in L2 than in L1 (97.5% vs. 95.7%). There were no other significant main effects or interactions (*ps* < 0.13; more details of the final model could be found in supplementary materials Table S3).

### Discussion

In Experiment 2, we again successfully demonstrated the semantic classifier congruency effect in L1, which in Experiment 2 was Chinese. However, in our language-switching experiment, we were not able to demonstrate the semantic classifier congruency effect for L2 English, while Experiment 1 showed a semantic classifier congruency effect for English when it was the L1 of participants. Yet, it is noteworthy that in the L2 English trials of Experiment 2, the numerical data pattern also showed 5 ms faster responses for semantically congruent classifier-noun pairs than for semantically incongruent pairs.

As regards language switching, Experiment 2 showed a reversed language dominance effect (faster responses in the non-dominant L2 than in L1), which could be taken as evidence for a role of proactive inhibitory control in language selection (cf. Declerck & Koch, 2023). That is, one could argue that the dominant language was proactively inhibited so that L1 responses became slower than L2 responses (see Declerck, 2020). In addition, we also found a smaller (rather than larger) language-switch cost in L1 than in L2, which represents a “reversed” asymmetrical switch cost that, however, is in line with a number of previous studies (Bonfieni et al., 2019; Declerck et al., 2015b; Zheng et al., 2020; for general review, see Gade et al., 2021). We will discuss this result further in the General Discussion.

Importantly, the semantic classifier congruence effect and language-switch cost did not interact with each other. Thus, Experiment 2 did not indicate an interplay between concepts/words activation and language switching. In other words, having the possibility to activate the language (by means of the classifier language) and the concept of the to-be-named word (by means of the classifier semantic meaning) in congruent trials did not significantly reduce the language-switch cost (although there was a numerical trend in the expected direction with the switch cost in semantically congruent trials being smaller than in incongruent trials 40 ms vs. 52 ms). However, one also has to consider that the semantic classifier congruence effect, specifically in L2, was relatively small in general.

## Experiment 3

The aim of Experiment 3 was to further examine the semantic classifier congruency effect in a bilingual, language-switching situation. In contrast to Experiment 2, in which classifiers were presented visually, Experiment 3 used auditorily presented classifiers. This change was motivated by the consideration that the small and not significant semantic classifier congruency effect for L2 in Experiment 2 could (at least partially) be due to the visual modality in which the classifiers were presented. On the one hand, participants might simply have ignored the visually presented classifier in some of the trials. On the other hand, an auditory processing of the classifier combined with a vocal response more closely resembles conversations in our daily life. More importantly, on a theoretical level one can argue that perceiving the classifier visually and naming the picture vocally might have led to cross-modal interference (Stephan & Koch, 2010, 2011, 2016; Schaeffner et al., 2016a, b; Schaeffner & Philipp, 2020). Bilingual participants might be burdened with such interference more heavily in the weaker language (i.e., L2 English) than in the dominant language. Thus, in order to rule out that the visual modality of the classifier had a negative effect on the semantic classifier congruence effect in L2, we replicated the basic design of Experiment 2 but presented the classifier auditorily in Experiment 3.

### Method

#### Participants

We recruited forty new Chinese (L1)-English (L2) bilingual participants studying at RWTH Aachen University (23 females, 17 males; 40 right-handed; mean age: M = 24.63 years old, SD = 2.35). They were asked to complete a questionnaire about the age of L2 acquisition, the self-rated language skills (7-point scale: 1 = “quite poor”, 7 = “highly proficient”, see Table 2) for English and reported results from proficiency online testing in a sentence completion task (http://www.itt-leipzig.de/static) in both Chinese and English. For the results of online testing, a paired-sample t-test showed a significant difference between the scores of L1 (91.85% accuracy) and L2 (56.90% accuracy), *t*(39) = 15.54, *p* <.001.

**Table 2:** Participants’ characteristics in experiment 3

Item	L2
AOA	8.62 (3.18)
Self-rating	
*Listening*	4.77 (1.37)
*Speaking*	4.31 (1.43)
*Reading*	5.28 (1.08)
*Writing*	4.62 (1.25)

*AOA* age of acquisition. The self-rated language skills were reported on a 7-point scale: 1 = “quite poor”, 7 = “highly proficient”. The value in the bracket represents the standard deviation

#### Materials and apparatus

**Fig. 2:**
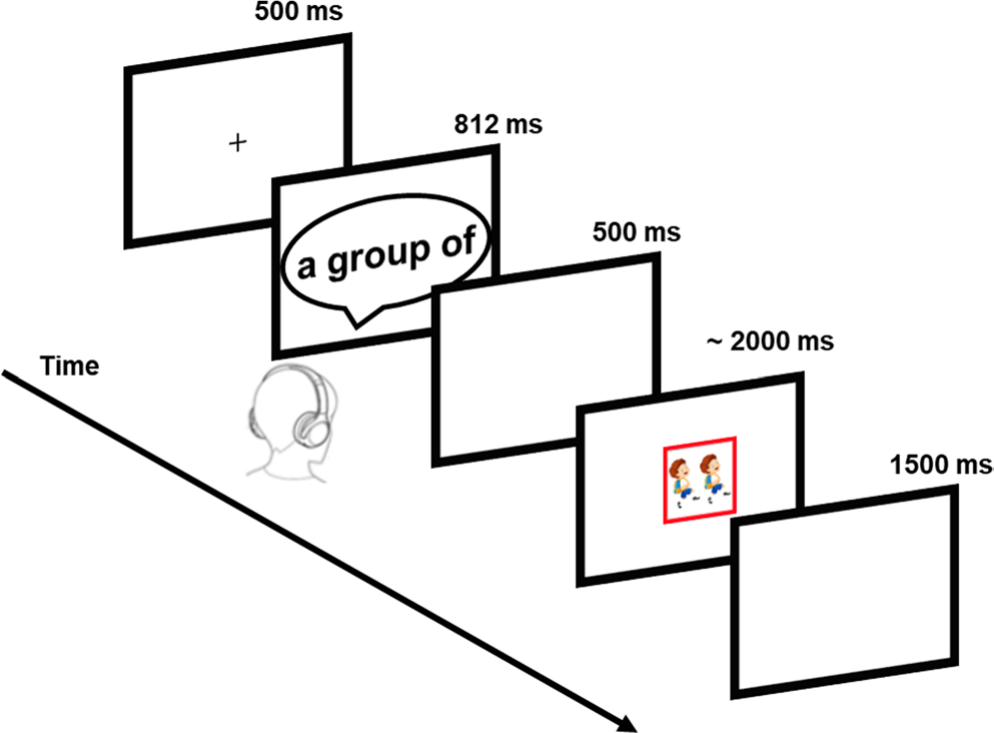
Experimental procedure of Experiment 3. *Note:* Because the number of syllables for classifiers was different between L1 and L2, the duration of the auditorily presented classifiers was variable. The average duration was 812 ms (range: 685 ms to 997 ms)

The materials and apparatus were identical to those used in Experiment 2 except that classifier were now presented as audio files (see Fig. 2). We used a text-to-speech system in Google Cloud to generate realistic-sounding human voices (duration: M = 812 ms, SD = 84 ms; format: wav; speed rate: normal; sampling rate: 48000 Hz). The voice in all audio files was confirmed to be similar based on careful inspection by the experimenter. The time range of all classifiers was 685 ms to 997 ms. The duration of the Chinese classifier was shorter than those of the English classifier (736 ms vs. 889 ms; *t* = −13.80, *p* <.001) because the Chinese classifiers have fewer syllables than the English ones (2.00 vs. 3.25; *t* = − 13.84, *p* <.001). Yet, please note that the English classifier always had the form of ‘a X of’ so that the first and the last syllable did not contain any specific meaning, so that the prediction of semantically congruent nouns could start earlier as indicated by the length of the audio file and, thus, more comparable to the Chinese classifier.

#### Procedure

The procedure was the same as in Experiment 2 except that the classifier was presented through headphones.

#### Design

We used a 2 × 2 × 2 within-subjects designs with the independent within-subject variables semantic congruence (noun semantically congruent vs. incongruent to the classifier), language (L1 vs. L2), and language sequence (repeat vs. switch).

#### Data analysis

Data analysis was the same as in Experiment 2. For the maximal model, semantic congruence, language, and language sequence were fixed effects, participants and items were random effects. Semantic congruence, language and sequence were included as a by-subject slopes and by-item slopes. For RT analysis, RTs were log-transformed used as dependent variable (DV). Two-level categorical predictors were coded as −0.5 and 0.5. For accuracy analysis, response type (correct trials coded as 1; error trials coded as 0) was the dependent variable (DV).

### Results

Using the same criteria as in Experiment 2, we excluded 10.69% of trials for RT analysis and 7.43% of trials for accuracy analysis.

#### Reaction time

The main effect of semantic congruence was significant, *β* = 0.06, *SE* = 0.003, *t* = 19.11, *p* <.001, with 58 ms shorter reaction times in trials with congruent classifier-noun combinations compared to trials with incongruent combinations (954 ms vs. 1012 ms). The main effect of language was significant, *β* = − 0.06, *SE* = 0.01, *t* = −4.51, *p* <.001, with faster response in L2 than in L1 (953 ms vs. 1012 ms). Like in Experiment 2, the main effect of language sequence was also significant, *β* = 0.05, *SE* = 0.003, *t* = 14.21, *p* <.001, showing better performance in repeat trials than in switch trials (959 ms vs. 1007 ms) and, thus, a switch cost of 48 ms.

The interaction of semantic congruence and language was significant, *β* = − 0.07, *SE* = 0.01, *t* = −11.31, *p* <.001. Follow-up analyses showed faster responses in congruent trials than in incongruent trials in both L1 (965 ms vs. 1060 ms), *β* = − 0.10, *SE* = 0.004, *z* = −21.35, *p* <.001, and L2 (942 ms vs. 964 ms), *β* = − 0.03, *SE* = 0.004, *z* = −5.55, *p* <.001, yet, the size of the semantic classifier congruency effect was larger for L1 than for L2 (95 ms vs. 22 ms).

Additionally, the interaction of language and language sequence was significant, *β* = 0.02, *SE* = 0.01, *t* = 3.47, *p* <.001. Follow-up analyses showed a significant switch cost in L1 (994 ms vs. 1031 ms), *β* = − 0.04, *SE* = 0.01, *z* = −7.55, *p* <.001, and in L2 (924 ms vs. 982 ms), *β* = − 0.06, *SE* = 0.01, *z*= −12.59, *p* <.001, but the cost was smaller in L1 than in L2 (37 ms vs. 58 ms), similar to Experiment 2.

Finally, unlike in Experiment 2, the theoretically relevant interaction of semantic congruence and language sequence was significant, too, *β* = 0.02, *SE* = 0.01, *t* = 3.55, *p* <.001. Follow-up analyses showed that a significant language-switch cost occurred in both congruent (repeat vs. switch: 936 ms vs. 971 ms, *β* = − 0.04, *SE* = 0.01, *z* = −7.53, *p* <.001) and incongruent trials (repeat vs. switch: 981 ms vs. 1042ms, *β* = − 0.06, *SE* = 0.01, *z* = −12.58, *p* <.001), but the predictability in congruent trials led to smaller switch cost in these trials as compared to incongruent trials (35 ms vs. 61 ms). There was no other significant interaction (*p* =.81; more details of the final model could be found in supplementary materials Table S4).

#### Accuracy

The main effect of language was significant, *β* = 0.62, *SE* = 0.12, *z* = 5.06, *p* <.001, with higher accuracy in L2 than in L1 (97.1% vs. 95.1%). The main effect of language sequence was also significant, *β* = 0.73, *SE* = 0.09, z = 8.50, *p* <.001, with higher accuracy in repeat trials than in switch trials (97.3% vs. 94.9%).

The interaction of language and language sequence was significant, *β* = 0.44, *SE* = 0.17, *z* = 2.53, *p* =.01. Follow-up analyses showed higher accuracy in L1 repeat trials than in L1 switch trials (96.3% vs. 94.0%), *β* = 0.52, *SE* = 0.10, *z* = 5.04, *p* <.001; and in L2 repeat trials and L2 switch trials (98.3% vs. 95.9%), *β* = 0.95, *SE* = 0.13, *z* = 6.84, *p* <.001, indicating significant language-switch cost that were smaller in L1 (2.3%) than in L2 (2.4%). There were no other main effects or interactions (*ps* > 0.22; more details of the final model could be found in supplementary materials Table S5).

### Discussion

In Experiment 3, we replicated the semantic classifier congruency effect in L1, a reversed language dominance effect and a smaller language-switch cost in L1 compared to L2. Furthermore, we had two additional, new findings in Experiment 3. First, the observed 22 ms semantic classifier congruency effect in L2 English was now clearly significant (albeit also significantly smaller than in L1 Chinese). Second, the language-switch cost was modulated by semantic classifier congruency in a way that a smaller switch cost was found in congruent conditions compared with incongruent conditions. Thus, Experiment 3 indicated that being able to activate both the language and the concept of the response influences bilingual language control and reduced the language-switch cost (cf. Declerck et al., 2015a, b).

## General discussion

In this study, we explored whether classifier processing evoked prediction and thus modulated subsequent language production. More specifically, we examined the semantic classifier congruency effect and tested whether it also modulated language control during language switching. In Experiment 1, the semantic classifier congruency effect was established in a picture-naming task in an English monolingual experiment. In Experiment 2 with Chinese-English bilinguals, the semantic classifier congruency effect was significant in L1 (Chinese), while the 5 ms difference between congruent and incongruent classifier-noun pairs in L2 (English) was not significant. Finally, Experiment 3 again tested Chinese-English bilinguals but classifiers were presented auditorily (as compared to a visual presentation in Experiments 1 and 2). We observed a significant semantic classifier congruency effect in both L1 and L2. Yet, the effect was still substantially larger in L1 (Chinese) than in L2 (English). In addition, we found a smaller language switch cost in congruent conditions compared with incongruent conditions in Experiment 3 (and the same numerical tendency in Experiment 2), which demonstrates a modulation of language-switch cost by prediction. Finally, in Experiment 2 and 3, we also observed a reversed language dominance effect and a smaller language-switch cost in L1 than in L2 (i.e., a reversed asymmetrical switch cost). We will discuss these results in turn.

### Prediction and semantic classifier congruency effect

In our study, participants were presented with a classifier and given time to process it before naming a picture (i.e., producing a noun). By presenting the classifier first, we created a highly constrained context, which is known to trigger predictions (DeLong et al., 2005; Federmeier 2007; Staub et al., 2015; Wlotko & Federmeier, 2012). We hypothesized that prediction via classifier processing will influence language production, indicated by a semantic classifier congruency effect.

In both monolingual (Experiment 1) and bilingual situations (Experiments 2 and 3), the semantic classifier congruency effect was found. Both native English speakers and bilingual Chinese-English speakers were faster in semantically congruent trials than in semantically incongruent trials. This finding is, on the one hand, in line with previous studies (Wang et al., 2006, Experiment 2; Zhang & Liu, 2009, Experiment 1; Huang & Schiller, 2021; Wang et al., 2025a, b; but see Wang et al., 2019 & Y. Wang et al., 2025a, b) which also demonstrated a semantic classifier congruency effect. On the other hand, this result confirmed that prediction via classifier processing influenced language production, consistent with a previous, monolingual study (Grisoni et al., 2024). To explain this effect, one can argue that faster language production resulted from language and concepts/words activation based on reading/hearing classifiers (Huettig et al., 2010; Mitsugi, 2020). That is, predicting semantically congruent pictures after reading/hearing classifiers leads to a pre-activation of corresponding concepts/words for the following picture-naming task.

Given that a previous study indicated that classifiers with a wide variety of semantically congruent nouns elicited a larger negativity in event-related potentials than classifiers with a limited number of semantically congruent nouns (Qian, & Garnsey, 2016), one might argue that the semantic classifier congruence effect observed in our study should also differ between nouns with many vs. few congruent classifiers (e.g., “a packet of” or “a pair of” vs. “a bouquet of”). Although our study can provide anecdotal evidence for this claim (e.g., in all experiments, the semantic classifier congruency effect was larger for the classifier “a bouquet of” than for the classifier “a packet of”; however, only in Experiment 2 and 3, a larger semantic classifier congruency effect was found for the classifier “a bouquet of” than for the classifier “a pair of”), the study was not designed in a way that allows a systematic analysis including the number of semantically congruent nouns per classifier as additional variable.

### Language- and modality-specific influences during prediction

Even though we found a semantic classifier congruency effect in English in Experiment 1 (i.e., with native English speakers), this was not always the case in the bilingual experiments when English was the L2 of the participants. More specifically, in Experiment 2, bilinguals responded faster in congruent trials than in incongruent trials in L1 Chinese but not significantly so in L2 English, which might imply less or even no prediction during L2 processing (cf. Ito et al., 2017; Mitsugi & Macwhinney, 2016). However, in Experiment 3, we observed a significant semantic classifier congruency effect in both L1 Chinese and L2 English, which is consistent with those studies showing a prediction effect also during L2 processing (Chun & Kaan, 2019; Dijkgraaf et al., 2017; Foucart et al., 2014).

Despite this between-experiment difference regarding the semantic classifier congruency effect in L2, both Experiment 2 and 3 showed a smaller semantic classifier congruency effect in L2 English as compared to L1 Chinese, indicating a weaker prediction effect in L2 than L1. We suggest that this difference between L1 and L2 in Experiments 2 and 3 is mainly caused by a difference in language proficiency (i.e., a different prediction effect in L1 and L2) and, thus, represents a quantitative rather than a qualitative difference.

The reviews of Kaan (2014) and Schlenter (2023) also summarize that there is quantitative difference for prediction in L1 and L2 but no qualitative difference. Further, Martin et al. (2013) found that predictions during L2 comprehension do not occur to the same extent as they do during L1 comprehension. The authors suspected that the weaker prediction during L2 comprehension resulted from slower language processing in L2 as compared to L1 (see also Dijkgraaf et al., 2017; Momenian et al., 2024). Further research, thus, should investigate whether a longer time interval between the classifier presentation and noun production could possibly lead to a larger semantic classifier congruency effect, specifically for L2.

In addition to language-specific influences, our study also showed modality-specific influences on the semantic classifier congruency effect. More specifically, the classifier congruency effect in L2 was non-significant in Experiment 2, in which the classifiers were presented visually, but reached significance in Experiment 3, in which the classifiers were presented auditorily. That is, participants saw a visual classifier in Experiment 2 but heard an auditory classifier in Experiment 3, while they responded vocally in both experiments. This difference is important as, on the one hand, participants in Experiment 3 had to process the classifier, while they could have avoided to do so in Experiment 2 by simply not reading the classifier. On the other hand, previous research has shown that sensory-motor modality compatibility matters in language processing as participants switched between linguistic tasks more efficiently when the input-output modality combinations were compatible (i.e., auditory-vocal and visual-manual) as compared to incompatible (i.e., auditory-manual vs. visual-vocal; Schaeffner et al., 2016a, b; Schaeffner & Philipp, 2020). According to Morton’s logogen model (1969) there are specific feedback loops that directly connect vocal output and auditory input on the one hand and manual output and visual input on the other hand. These connections might provide preferred processing pathways linking vocal output to auditory input and manual output to visual input. For the present study, this idea could be transferred to suggesting a closer connection and thus potentially also a larger influence of prediction when the vocal production was preceded by auditory input.

### The influence of prediction on Language switching

In Experiment 3, the language-switch cost was significantly smaller in congruent trials than in incongruent trials, suggesting that prediction during classifier processing modulated language control in language switching. To account for this effect, we argue that classifier presentation elicited predictions with respect to both the relevant language and the relevant concept of the to-be-produced word. That is, the classifier most likely primed the relevant language, acting as a language cue (albeit the explicit language cue was presented only simultaneously with the picture). Previous studies (e.g., Liu et al., 2020; Verhoef et al., 2009) recognized the role of the language cue in language switching. H. Liu and colleagues (2020) suggested that a language cue could be used to update and maintain goal-relevant information during language switching. By seeing or hearing the classifier, the corresponding language might already have been prepared/activated, leading to an advantage to perform in this language. Additionally, being able to activate both the language and the concepts was already demonstrated to reduce language-switch cost in a study by Declerck and colleagues (2015). We therefore suggest that in the present study the possibility to predict both language and concepts/words also influenced language control and led to a reduction of language-switch cost.

Models of bilingual language control assume that between-language interference is resolved at presumably two different levels. One of them is a rather global language-related level (either language schemas, cf. Green, 1998, or language tags, cf. Declerck et al., 2015a, b), the other a word-specific level (i.e., lemma level). The external input that, in the present study, was provided by the classifier now provides the opportunity to start interference resolution on the language-related level, as the language is already primed/activated by the classifier language. According to previous studies on prediction through production systems, the linguistic levels of words—meaning, grammar, and sound—are activated either sequentially (Pickering & Gambi, 2018) or in parallel (Pickering & Strijkers, 2024). Therefore, we propose that in congruent trials, interference resolution at the lemma level may begin as the classifier is processed, enabling the prediction of upcoming concepts or words. This prediction, combined with the activation of the corresponding target language, supports interference resolution at the lemma level.

As the language of the response is already primed by the classifier language, prediction might already be language-specific. The reduction of language-switch cost based on semantically congruent classifier-noun combinations might hint toward a language-specific prediction. Further, considering theories that base prediction on the production system (Dell & Chang, 2014; Federmeier, 2007; Kuperberg & Jaeger, 2016; Lelonkiewicz et al., 2021; for reviews, see Pickering & Garrod, 2007, 2013; Pickering & Gambi, 2018) might also speak in favor of predictions going beyond the abstract concept level into the word level and maybe even further than that (i.e., production).

### Language dominance and reversed asymmetrical Language switch cost

In both Experiments 2 and 3, a reversed language dominance effect (i.e., better performance in L2 than in L1) and a reversed asymmetrical language-switch cost (i.e., smaller switch cost in L1 than in L2) were found. The reversed language dominance effect is typically explained by a general and proactive inhibition of L1 (see, e.g., Declerck, 2020, Declerck & Koch, 2023) in order to facilitate L2 production. With a strong inhibition of the more dominant language (which in general is the L1), L2 can become functionally more dominant for the time of a specific situation (i.e., a language-switching experiment). This might in turn also explain a larger language-switch cost for L2 (the currently more dominant language) compared to L1 (the currently less dominant language). Consequently, a reversed language dominance effect could come along with a reversed language-switch cost asymmetry, as observed in our study. This observation is in line with a number of previous studies (e.g., Bonfieni et al., 2019; Zheng et al., 2020; for a meta-analysis see Gade et al., 2021). Yet, in a meta-analysis by Gade et al. (2021), there was no strong evidence suggesting that L1 slowing is systematically related to the presence or absence of a switch cost asymmetry. Thus, we are cautious about this explanation. Future research will need to examine the relation of language dominance and reversed asymmetrical switch cost in more details.

Another explanation for the observed data pattern (i.e., a larger L2 than L1 switch cost) could arise from predictions via classifier processing in our experiment. Since the classifiers in our experiments were specifically designed to trigger predictions and the language of the classifier consistently matched the response language, it served as a naturalistic cue. Such a naturalistic cue might be a stronger cue compared to color-language cues (which are arbitrary and have to be learned by participants) or even national flags used in many previous studies (e.g., Costa, 2004; Verhoef et al., 2009). In the present experiment, thus, we suppose that hearing a classifier in one language already primed or activated that language. As we observed a larger prediction effect in L1 than in L2, we additionally suppose that the language activation was more pronounced for L1 than for L2. This activation difference might now have a critical importance in language-switch trials. In a language-switch trial, the irrelevant and thus inhibited language from the preceding trial needs to be reactivated. When this reactivation can be started during classifier processing and L1 is more strongly (re-)activated than L2, we also expect a larger switch-cost decrease for L1 than for L2, leading to a smaller switch cost for L1 as compared to L2. Thus, the reversed language-switch cost asymmetry is perfectly in line with the larger prediction effect for L1 than for L2 and further supports an interplay of prediction and language control in language switching.

### Prediction rather than integration?

In the present study, we considered the semantic classifier congruency effect as an empirical marker of prediction that occurs during language processing (cf. Ryskin & Nieuwland, 2023). Importantly, we did not focus on precise predictions (see Ito et al., 2016; Lelonkiewicz et al., 2021) because precise lexical predictions are rare in daily life (Luke & Christianson, 2016). Rather, we suppose that prediction takes place whenever we process information and leads to a pre-activation of possibly following lexical information. Such a view is for example supported by anticipatory brain activity before the onset of the following lexical information (Antoine et al., 2024; see also Ferreira & Chantavarin, 2018).

However, the present study cannot clearly distinguish between prediction as a top-down process leading to a pre-activation of concepts/words and integration as a bottom-up process. That is, faster word production for semantically congruent classifier-noun phrases as compared to incongruent classifier-noun phrases in our study could also be explained by a faster integration of the to-be-produced noun into a classifier-noun phrase as soon as the picture appears (i.e., bottom-up processes). Integration hereby refers to the process of connecting new lexical information into previously established or contextual frameworks in a bottom-up manner (cf. Gernsbacher, 1991; Kintsch & van Dijk, 1978). That is, new input that is coherent with the preceding context can be integrated more quickly than incoherent input, leading to faster comprehension during language processing (Hagoort et al., 2004). Thus, with respect to the present study, one may argue that congruent concepts are more coherent with the preceding context in the congruent condition, and thus faster integrated, as compared with incongruent ones.

Although previous research has aimed to differentiate prediction from integration (Ferreira & Chantavarin, 2018; Mantegna et al., 2019; Nieuwland et al., 2019), it remains challenging to determine whether processing benefits arise primarily from predicted input or from facilitated integration with a matching context (DeLong et al., 2005; Federmeier, 2007; Ryskin & Nieuwland, 2023). However, as prior eye-movement studies (Huettig et al., 2010; Mitsugi, 2020) suggest that processing a classifier activates related concepts or words (i.e., participants already looked towards a congruent picture before the noun was presented), we cautiously propose that prediction plays a significant role in our findings, though integration processes may also occur.

Additionally, there are two aspects of the present study that speak in favor of prediction rather than integration. First, it is important to note that the observed semantic classifier congruency effect in our study was measured in the RT of a language production task. In contrast, integration is typically explored in language comprehension tasks, in which the lexical input is presented, making bottom-up influences more likely. Of course, integration could have taken place on a semantic, conceptual level in the present study, so that the concept that was presented as visual object was integrated in the classifier-noun phrase, which in turn was beneficial for the production of the correct response in the correct language. However, if semantic integration alone played a role in the current study, the facilitation effect resulting from integration should be language-independent. This was clearly not the case in the present study as we observed a substantially larger effect in L1 than in L2. This result cannot be fully explained by bottom-up integration. Instead, we suppose that prediction occurred during the processing of the classifier, which led to a pre-activation of both language and concepts before the picture was presented.

Second, the results of the present study showed that language-switch costs were smaller in the classifier congruent condition than in the classifier incongruent condition. From both language- and task-switching research we have evidence that a long preparation time can reduce switch costs (for a direct comparison see Graham & Lavric, 2021; Lavric et al., 2019). That is, a longer time to prepare for a switch allows the pre-activation of the upcoming language or task, so that between-language interference as well as interference between tasks can already be resolved (at least to some degree). The reduction of language-switch costs in the present study thus indicates that participants could use the time interval between classifier and noun to prepare and to start interference resolution. Put differently, in order to observe a reduction of switch costs, participants needed to pre-activate the upcoming language and the upcoming concept (cf. Declerck et al., 2015a, b), which corresponds to prediction rather than integration.

### Theoretical implications and conclusion

In conclusion, we found that prediction during classifier processing influenced subsequent language production in both L1 and L2. However, the effect was substantially larger in L1. Thus, we conclude that prediction is more pronounced in the native language than in a foreign language.

Finally, the present study also showed that prediction during classifier processing reduced language-switch costs. This finding implies that when both the language and the concepts/words of a to-be-named picture can be predicted, between-language interference can be reduced so that language production is less affected by language switching. Thus, prediction is not only beneficial for subsequent word production but also for bilingual language control.

## Supplementary Information

Below is the link to the electronic supplementary material.

Supplementary Material 1

Supplementary Material 2

## Data Availability

The data that support the findings of this study are openly available in PsychArchives at http://dx.doi.org/10.23668/psycharchives.13149.

## Appendix

**Table 3:** Classifier-noun pairs in Experiment 1

Classifier		Congruent		Incongruent	
English	Chinese	English	Chinese	English	Chinese
a bottle of	一瓶	milk	牛奶	noodle	面条
a bouquet of	一束	flower	花	bread	面包
a bowl of	一碗	noodle	面条	student	学生
a bunch of	一串	grape	葡萄	milk	牛奶
a bundle of	一捆	hay	干草	snow	雪
a coil of	一卷	wire	电线	ant	蚂蚁
a crew of	一队	fireman	消防员	rain	雨
a cup of	一杯	tea	茶	brick	砖
a dish of	一盘	ice cream	冰激凌	shoe	鞋
a drop of	一滴	rain	雨	flower	花
a grain of	一粒	wheat	麦子	book	书
a group of	一群	student	学生	glue	胶水
a jar of	一罐	jam	果酱	hay	干草
a nest of	一窝	ant	蚂蚁	tissue	纸巾
a packet of	一包	tissue	纸巾	fireman	消防员
a pair of	一双	shoe	鞋	tea	茶
a pile of	一堆	brick	砖	honey	蜂蜜
a row of	一排	book	书	ice cream cream	冰激凌
a set of	一套	stamp	邮票	wheat	麦子
a shovel of	一铲	snow	雪	grape	葡萄
a slice of	一片	bread	面包	coin	硬币
a spoon of	一勺	honey	蜂蜜	stamp	邮票
a stack of	一摞	coin	硬币	wire	电线
a tube of	一管	glue	胶水	jam	果酱

**Table 4:** Classifier-noun pairs in Experiment 2 and 3

Classifier		Congruent		Incongruent	
English	Chinese	English	Chinese	English	Chinese
a basket of	一篮	apple	苹果	coin	硬币
a bottle of	一瓶	milk	牛奶	shoe	鞋
a bouquet of	一束	flower	花	bread	面包
a bowl of	一碗	noodle	面条	card	纸牌
a box of	一箱	candy	糖果	student	学生
a bundle of	一捆	hay	干草	milk	牛奶
a coil of	一卷	wire	电线	ant	蚂蚁
a crew of	一队	fireman	消防员	candy	糖果
a cup of	一杯	tea	茶	brick	砖头
a deck of	一副	card	纸牌	tea	茶
a drop of	一滴	water	水	flower	花
a grain of	一粒	wheat	麦子	apple	苹果
a group of	一群	student	学生	glue	胶水
a jar of	一罐	jam	果酱	hay	干草
a nest of	一窝	ant	蚂蚁	tissue	纸巾
a packet of	一包	tissue	纸巾	fireman	消防员
a pair of	一双	shoe	鞋	water	水
a pile of	一堆	brick	砖头	jam	果酱
a set of	一套	stamp	邮票	wheat	麦子
a shovel of	一铲	snow	雪	honey	蜂蜜
a slice of	一片	bread	面包	wire	电线
a spoon of	一勺	honey	蜂蜜	stamp	邮票
a stack of	一摞	coin	硬币	noodle	面条
a tube of	一管	glue	胶水	snow	雪

## Footnotes

1 In the second pre-test, participants also performed an experiment with the same 2 × 2 × 2 (semantic congruence × word type × picture type) within-subjects design, but the combination of word and picture type was not fully counterbalanced across participants. Yet, a comparable significant main effect of semantic congruence was found with 24 ms faster responses in semantically congruent trials than in incongruent trials (779 ms vs. 803 ms, *β* = 0.03, *SE* = 0.01, *t* = 3.30, *p* =.002, for the full data set see 10.23668/psycharchives.12588).
