# Supplementary material for: The influence of prediction on bilingual language production: evidence from semantic classifier congruency
Source: Psychol Res. 2025 Nov 11;89(6):170. doi: 10.1007/s00426-025-02204-2 (PMC12605514; doi:10.1007/s00426-025-02204-2)
Supplement: Supplementary file 1 — Supplementary Material 1 [file 426_2025_2204_MOESM1_ESM.docx]

**ChatGPT have been used to correct some language errors. Additionally, its suggestions regarding word choice have been used as references for refining language usage. In the following section, “input” is the content being written in the ChatGPT and “output” is the content generating by ChatGPT.**

**Input:** make it sounds correct and clear: Since cognitive revolution in psychology kicked started in 1950s, we commonly agree on the mind functioning as an information-processing system, a mechanism allowing human perceiving and transforming information from surrounding world.

**Output:** Since the cognitive revolution in psychology began in the 1950s, it has become widely accepted that the mind functions as an information-processing system—a mechanism that enables humans to perceive and transform information from the surrounding world.

**Input:** does kicked started is correct here

**Output:** No, "kicked started" is not correct. The correct phrase is "kicked off" or simply "started". If you want to keep the idea of something being initiated energetically, you could say:

"Since the cognitive revolution in psychology kicked off in the 1950s, it has been widely agreed that the mind functions as an information-processing system—a mechanism that enables humans to perceive and transform information from the surrounding world."

**Input:** make it sounds correct and clear: While previous studies have focus on how the predictability of the following input influence monolingual and bilingual language processing (ref), less is focusing on how prediction itself affect language production during language switching.

**Output:** While previous studies have focused on how the predictability of upcoming input influences language processing in monolinguals and bilinguals (ref), less attention has been given to how prediction itself affects language production during language switching.

**Input**：make it sounds clear: So far, rich evidences have shown predictability of subsequent input impact following language processing. Question would naturally emerge about how this happens.

**Output:** So far, extensive evidence has demonstrated that the predictability of subsequent input influences language processing. This naturally raises the question of how this process occurs.

**Input：**make it clear and readable: Although these studies did not specify how many words are activated through prediction, previous research on language comprehension (Delong et al., 2005; Wlotko & Federmeier, 2012) suggests that comprehenders generate predictions by evaluating the likelihood of various possible outcomes. They dynamically adjust these probabilities as bottom-up input either confirms or disconfirm their predictions. Additionally, Staub et al. (2015) demonstrated that an activation-based race model could explain predictive processes in language production. The model suggested that multiple potential responses are activated in highly constrained contexts. These activated responses then independently "race" toward reaching a response threshold, ultimately determining the final output.

**Output:** Here’s a clearer and more readable revision of your paragraph:

Although these studies did not specify the number of words activated through prediction, previous research on language comprehension (Delong et al., 2005; Wlotko & Federmeier, 2012) suggests that comprehenders generate predictions by evaluating the likelihood of various possible outcomes. These probabilities are dynamically adjusted as bottom-up input either confirms or contradicts the predictions. Furthermore, Staub et al. (2015) showed that predictive processes in language production can be explained using an activation-based model. This model proposes that in highly constrained contexts, multiple potential responses are activated simultaneously. These responses then independently "race" to reach a response threshold, with the first to do so determining the final output.

**Input:** make it sounds formal, although these studies did not specify the fine grain aspects triggered by prediction (e.g., the number of words activated through prediction), previous research on language comprehension (Delong et al., 2005; Wlotko & Federmeier, 2012) suggests that comprehenders generate predictions by evaluating the likelihood of various possible outcomes.

**Output:** Although these studies did not specify the finer-grained aspects of prediction, such as the number of words activated, previous research on language comprehension (Delong et al., 2005; Wlotko & Federmeier, 2012) indicates that comprehenders generate predictions by assessing the likelihood of various potential outcomes.
