# Supplementary material for: The influence of prediction on bilingual language production: evidence from semantic classifier congruency
Source: Psychol Res. 2025 Nov 11;89(6):170. doi: 10.1007/s00426-025-02204-2 (PMC12605514; doi:10.1007/s00426-025-02204-2)
Supplement: Supplementary file 2 — Supplementary Material 2 [file 426_2025_2204_MOESM2_ESM.docx]

**Table S1**

Linear mixed-effect model for RT in Experiment 1

| Predictor |  | *β* | *SE* | | Contrast | | | |  |
| --- | --- | --- | --- | --- | --- | --- | --- | --- | --- |
|  |  |  |  |  | *t* | | *p* | |  |
| Fixed effects |  |  |  | |  | |  | |  |
| Intercept |  | 6.58 | .02 | | 261.66 | | < .001*** | |  |
| Semantic congruence | | .03 | .01 | | 3.81 | | < .001*** | |  |
| Word type |  | -.004 | .02 | | -.27 | | .79 | |  |
| Picture type |  | .001 | .01 | | .09 | | .93 | |  |
| Semantic congruence × word type | | -.00009 | .01 | | -.0008 | | .99 | |  |
| Semantic congruence × picture type | | .01 | .01 | | 1.78 | | .07 | |  |
| Word type × picture type | | .02 | .01 | | 2.88 | | .004** | |  |
| Semantic congruence × word type × picture type | | .01 | .01 | | .59 | | .55 | |  |
| Random effects | | *Variance* | *SD* | *Correlation* | | | | | |
| Subject | Intercept | .01 | .12 |  | |  |  | |  |
|  | Semantic congruence | .001 | .03 | -.33 | |  |  |  |  |
|  | Picture type | .0006 | .02 | -.19 | | .01 |  |  |  |
| Item | Intercept | .003 | .06 |  | |  |  |  |  |
|  | Semantic congruence | .001 | .04 | -.02 | |  |  |  |  |

**Table S2**

Linear mixed-effect model for RT in Experiment 2

| Predictor |  | *β* | | *SE* | | Contrast | | | | |
| --- | --- | --- | --- | --- | --- | --- | --- | --- | --- | --- |
|  |  |  |  |  |  | *t* | | *p* | | |
| Fixed effects |  |  | |  | |  | |  | | |
| Intercept |  | 6.93 | | .02 | | 287.20 | | < .001*** | | |
| Semantic congruence | | .02 | | .003 | | 5.94 | | < .001*** | | |
| Language |  | -.09 | | .01 | | -8.88 | | < .001*** | | |
| Sequence |  | .05 | | .01 | | 5.14 | | < .001*** | | |
| Semantic congruence × language | | -.03 | | .01 | | -3.92 | | < .001*** | | |
| Semantic congruence × sequence | | .01 | | .01 | | 1.69 | | .09 | | |
| Language × sequence | | .03 | | .01 | | 3.99 | | < .001*** | | |
| Semantic congruence × language × sequence | | -.02 | | .01 | | -1.65 | | .10 | | |
| Random effects | | *Variance* | | *SD* | *Correlation* | | | | | |
| Subject | Intercept | .02 | | .15 |  | |  | |  | |
|  | Language | | .002 | .05 | -.16 | |  | |  |  |
|  | Sequence | | .001 | .02 | -.11 | | .12 | |  |  |
| Item | Intercept | | .001 | .02 |  | |  | |  |  |
|  | Language | | .001 | .03 | .03 | |  | |  |  |
|  | Sequence | | .001 | .04 | -.25 | | -.14 | |  |  |

**Table S3**

Linear mixed-effect model for accuracy in Experiment 2

| Predictor | |  | *β* | | *SE* | | Contrast | | | | |
| --- | --- | --- | --- | --- | --- | --- | --- | --- | --- | --- | --- |
|  |  |  |  |  |  |  | *t* | | | *p* | |
| Fixed effects |  | |  | |  | |  | | |  | |
| Intercept |  | | -11.43 | | 1.47 | | -7.79 | | | < .001*** | |
| Semantic congruence | | | .20 | | .40 | | .50 | | | .62 | |
| Language |  | | 4.42 | | .51 | | 8.69 | | | < .001*** | |
| Sequence |  | | .33 | | .45 | | .73 | | | .47 | |
| Semantic congruence × language | | | .70 | | .80 | | .87 | | | .38 | |
| Semantic congruence × sequence | | | -.57 | | .80 | | -.72 | | | .47 | |
| Language × sequence | | | 1.36 | | .90 | | 1.51 | | | .13 | |
| Semantic congruence × language × sequence | | | -.37 | | 1.60 | | -.23 | | | .82 | |
| Random effects |  | | *Variance* | *SD* | | *Correlation* | | | | | |
| Item | Intercept | | 251.8 | 15.87 | |  | |  |  | |  |

**Table S4**

Linear mixed-effect model for RT in Experiment 3

| Predictor |  | *β* | | *SE* | | Contrast | | | | |
| --- | --- | --- | --- | --- | --- | --- | --- | --- | --- | --- |
|  |  |  |  |  |  | *t* | | *p* | | |
| Fixed effects |  |  | |  | |  | |  | | |
| Intercept |  | 6.86 | | .02 | | 309.23 | | < .001*** | | |
| Semantic congruence | | .06 | | .003 | | 19.11 | | < .001*** | | |
| Language |  | -.06 | | .01 | | -4.51 | | < .001*** | | |
| Sequence |  | .05 | | .003 | | 14.21 | | < .001*** | | |
| Semantic congruence × language | | -.07 | | .01 | | -11.31 | | < .001*** | | |
| Semantic congruence × sequence | | .02 | | .01 | | 3.55 | | < .001*** | | |
| Language × sequence | | .02 | | .01 | | 3.47 | | < .001*** | | |
| Semantic congruence × language × sequence | | -.003 | | .01 | | -.25 | | .81 | | |
| Random effects | | *Variance* | | *SD* | *Correlation* | | | | | |
| Subject | Intercept | .02 | | .125 |  | |  | |  | |
|  | Language | | .002 | .05 | -.20 | |  | |  |  |
| Item | Intercept | | .002 | .05 |  | |  | |  |  |
|  | Sequence | | .002 | .05 | -.10 | |  | |  |  |

**Table S5**

Linear mixed-effect model for accuracy in Experiment3

| Predictor |  | *β* | *SE* | | Contrast | | | | |
| --- | --- | --- | --- | --- | --- | --- | --- | --- | --- |
|  |  |  |  |  | *z* | | *p* | | |
| Fixed effects |  |  |  | |  | |  | | |
| Intercept |  | -3.53 | .12 | | -29.43 | | < .001*** | | |
| Semantic congruence | | .03 | .09 | | .33 | | .74 | | |
| Language |  | .62 | .12 | | 5.06 | | < .001*** | | |
| Sequence |  | .73 | .09 | | 8.50 | | < .001*** | | |
| Semantic congruence × language | | -.08 | .17 | | -.46 | | .64 | | |
| Semantic congruence × sequence | | .17 | .17 | | .98 | | .33 | | |
| Language × sequence | | .44 | .17 | | 2.53 | | .01** | | |
| Semantic congruence × language × sequence | | -.42 | .35 | | -1.21 | | .23 | | |
| Random effects | | *Variance* | *SD* | *Correlation* | | | | | |
| Subject | Intercept | .47 | .68 |  | |  | |  | |
|  | Language | .17 | .41 | -.04 | |  | |  |  |
